# Mediating role of social disengagement and loneliness in the nexus between functional health and mental well-being in older individuals

**DOI:** 10.1038/s41598-024-66919-9

**Published:** 2024-07-14

**Authors:** Papai Barman, Dil Bahadur Rahut, Rakesh Mishra

**Affiliations:** 1 International Institute for Population Sciences, Mumbai, 400088 India; 2 Asian Development Bank Institute, Tokyo, Japan; 3 UNICEF, New Delhi, India

**Keywords:** Depression, Functional health, Social disengagement, Loneliness, Living arrangement, LASI, India, Human behaviour, Risk factors, Geriatrics, Public health, Quality of life

## Abstract

Few studies have focused sufficiently on the intricate link between functional health and depression among older people aged 60 and above in India. Therefore, the current study investigates the association between functional health and depression among older Indian adults through the mediating role of social disengagement and loneliness and the moderating role of living arrangements using recent data from the Longitudinal Aging Study in India (LASI: 2017–2018). Composite International Diagnostic Interview-Short Form (CIDI-SF) for depression, the Activities of Daily Living (ADL) and Instrumental Activities of Daily Living (IADL) for functional health, and the indoor/outdoor activities, visits, and religious events for social disengagement were used. The feelings of loneliness and living arrangements were measured using single-item questions and surveys/interviews of household members. Bivariate analysis, logistic regression, and a Partial Least Squares-Structural Equation Model were adopted. The results show that older persons with functional health had 1.85 times higher odds of depression; similarly, those not engaging in social activities and experiencing loneliness were more likely to feel depressed. Living with someone was negatively linked to depression. A significant moderation by living arrangements in the functional health-depression relationship was also observed. The results also indicate significant mediating roles of social disengagement and loneliness, with 22.0% and 3.08% mediation effects, respectively. Therefore, this study recommends the provision of housing and social interaction among older people.

## Introduction

The mental and physical well-being of older individuals is of concern in public health policy and practice, given their association with increased morbidity, disability, and mortality. Among the older population, depression arises due to mental illness, characterised by long-term sadness, diminished interest in once-enjoyed activities, and difficulties with routine tasks. It leads to decreased self-worth and physical infirmity and increased suicidal thoughts^1,2^. The World Health Organization (WHO) estimates that approximately 280 million people worldwide suffer from depression^3^. Depressive symptoms are linked to suicide risk and an increased mortality risk in individuals with high cumulative mean depression scores. Studies have examined linkages between physical and mental well-being among older individuals in the past two to three decades^4^. However, the underlying association still needs to be more adequately explored in rapidly ageing countries like India. According to the Men and Women Report 2022 by the Ministry of Statistics & Programme Implementation, India's older population aged 60 and above is expected to increase from 101.5 million in 2011 to 227.4 million by 2031 due to an unconventional demographic transition^5^. The recent round of the Longitudinal Ageing Survey of India (LASI: 2017–18) reported that nearly one in every four older people (aged 60 and above) had reported depressive symptoms^6^. The empirics suggest that the burgeoning older population in India will encounter two-fold challenges stemming from functional health brought by ageing and nucleating families. Under such inevitable demographic change, understanding the health challenges among older adults becomes pertinent, especially mental health issues. In reality, it is vital to understand and differentiate the significance of the intermediary elements that have an impact on the intricate interaction between functional health and mental well-being. There is, however, a greater need to disentangle the roles of these intermediary factors as mediators and moderators between depression and functional health.

A series of studies have found an association between functional health and mental health issues among older individuals and explored pathways through which this relationship operates^7–9^. Furthermore, factors such as social engagement/disengagement, religiosity, loneliness, and living arrangements are also acknowledged as significant factors in understanding depression in older individuals with functional health^10,11^. However, few studies have assessed the actual influence of some of these factors as mediators in understanding the complex interplay between functional health and depression^9,12^. Chao^13^ examined social support moderating the relationship between functional difficulty and depression. Another study by Benka and colleagues^8^ argued that emotional support moderates the relationship between functional disability and depression among older adults with rheumatoid arthritis. Among several other factors, health-promoting behaviours and ongoing emotional, behavioural, and cognitive efforts for maintaining good health moderate the relationship between functional disability and depression^14^.

In India, significant efforts have been made to explore the factors contributing to the complex relationship between functional difficulty and depression^15^. However, very few studies have attempted to understand the pathways through which these factors mediate or moderate the relationship between functional health and depression. Muhammad and Maurya^16^ explored the role of social participation as a moderating factor influencing the association between functional difficulty and depression. In their study, researchers investigated the impact of social participation on the association between functional health and depression. However, the relationship between functional health and depression is not merely moderated by social participation but is, in fact, more accurately mediated by social engagement or disengagement. Banerjee and Boro^12^ noted a moderating influence of sleep quality and functional limitations on depression among older Indians.

In recent years, researchers and policymakers have paid increasing attention to functional health, among the inevitable consequences of ageing. Studies have identified that the coexistence of physical disabilities with ageing is associated with adverse emotional outcomes, such as depression and decreased life satisfaction^17–20^. Functional health has been extensively studied, highlighting how older individuals may become dependent on others due to physical limitations that hinder basic activities, increasing reliance on others and contributing to depression, resulting in depression^21–25^. Furthermore, it is also reported that individuals with functional health often experience social isolation and reduced social support, which can increase the risk of depression (based on their separate relationships with each other)^26,27^.

Social disengagement, isolation, and loneliness are frequently associated with ageing and are often linked with mental health issues. Individuals may experience fewer social interactions and reduced engagement with their social networks as they age. This can lead to feelings of isolation and loneliness, negatively impacting mental well-being^28–30^. The term “social disengagement” refers to fewer interactions with family members, friends, neighbours, office colleagues, and others^31^. On the other hand, “loneliness” is a more subjective experience that results from a perceived lack of social interaction^30^. People who experience social disengagement due to physical disabilities normally engage in fewer social activities and interactions^29,32^. Studies have also frequently linked functional health with social isolation and loneliness. Thompson and Heller^33^ noted that functional health negatively impacts social interaction. Based on the previous research and the trajectory of relationships among functional health, psychological well-being, and social disengagement, we have assumed a strong mediating role of social disengagement between functional health and depression. This theory provides insight into how and why functional health may result in mental health issues. Essentially, a chain of interrelated factors exists wherein functional health affects an individual’s social engagement or disengagement, which in turn impacts their mental well-being^34^.

The living arrangements of older individuals have a notable effect on their mental health. Co-habitation with a spouse and adult children is commonly linked to contentment, while living alone can lead to feelings of despondency^35^. In India, households that involve multiple generations, including spouses and adult children, are preferred due to cultural and traditional values. Any disruption or modification to these living arrangements may have a negative impact on one’s psychological health and overall well-being^36^. Shobhit et al.^37^ reported that in India, older people living alone were more likely to be depressed. Likewise, research from various countries has consistently found that living alone increases the risk of depression compared to living with family, friends, and relatives^2,38–41^.

In light of the above, the present study investigates the association between functional health and depression among older individuals in India. More specifically, it focuses on the possible influence of social disengagement and loneliness and the role of living arrangements on the pathways of association. As research into older adults’ health and living situations grows, one area that has yet to receive adequate attention is the possible link between functional health, depression, and social disengagement or loneliness. Additionally, the role of living arrangements in this relationship has been overlooked. However, understanding the impact of living arrangements on the relationship between functional health, social disengagement, loneliness, and depression is crucial. We may better understand the underlying association by exploring this neglected area. The first wave of Longitudinal Aging Study (2017–2018) in India (LASI), a nationally representative survey, allows us to fill gaps in current research and provide vital insights into the complex relationship between functional health and mental well-being in older individuals. The study hopes to establish targeted interventions that improve this vulnerable population’s overall quality of life. By examining social disengagement, loneliness, living arrangements, and social support separately and together, the research will provide a more comprehensive understanding of the causal relationship between these factors and their impact on mental health.

## Results

### Descriptive statistics

The data presented in Table 1 depict the distribution of the sample population based on their socio-economic characteristics. The sample size consisted of 31,902 individuals. The age groups were diverse, with the majority (32.18%) in the 60–64-year-old category, followed by the 65–69-year-old age group (28.04%). The gender distribution was almost equal, with 51.92% female and 48.08% male respondents. Interestingly, a significant proportion of respondents (63.36%) were currently married, while 33.99% were widowed. It was observed that 53.89% of the sample had no education, and only 15.08% had completed 10 years or more of education. Hinduism was the dominant religion among the respondents (73.01%), and 38.04% belonged to Other Backward Classes (OBC). The majority (66.09%) of the participants resided in rural areas, and approximately 20% fell into each category of the MPCE quintile (36%).

**Table 1:** Sample distribution of the study population by the respondent’s socio-economic characteristics in India, LASI, 2017–18.

Background characteristics	Percentage	Sample
Age category (Years)		
60–64 year	32.18	10,267
65–69 years	28.04	8944
70–74 years	18.22	5811
75 + years	21.57	6880
Sex		
Female	51.92	16,562
Male	48.08	15,340
Marital status		
Currently married	63.36	20,212
Widowed	33.99	10,845
Others	2.65	845
Education		
No education	53.89	17,191
Less than 5 years	12.02	3836
5–9 years completed	19.01	6065
10 years or more	15.08	4810
Religion		
Hindu	73.01	23,292
Muslim	11.70	3731
Christian	10.01	3194
Others	5.28	1685
Caste		
Scheduled Caste (SC)	16.17	5157
Scheduled Tribes (ST)	16.72	5334
Other Backward Class (OBC)	38.04	12,137
Others	29.07	9274
MPCE quintile		
Poorest	20.63	6580
Poorer	20.60	6573
Middle	20.38	6502
Richer	19.62	6259
Richest	18.77	5988
Currently working		
No	70.62	22,529
Yes	29.38	9373
Place of residence		
Rural	66.09	21,085
Urban	33.91	10,817
Chronic diseases		
No	45.70	14,579
Yes	54.30	17,323
Total	100.00	31,902

Source: computed using LASI (2017–18) data file.

**Figure 1:**
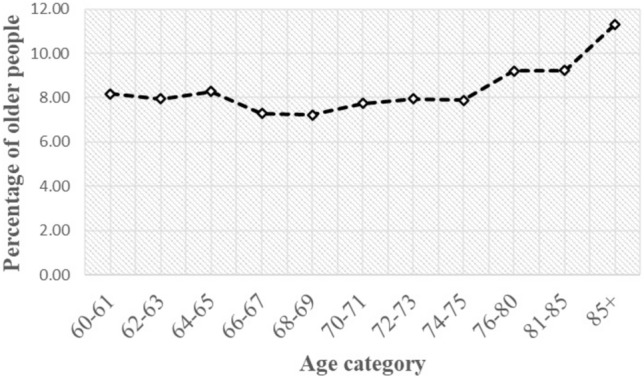
Sex-adjusted prevalence of depression by respondent age in India, LASI, 2017–18. Source: computed using LASI (2017–2018) data file.

Figure 1 shows the systematic increase in the sex-adjusted prevalence of depression with advancing age. The trend is consistent across all age groups, with the prevalence rising from 8.17% in the 60–61-year-old age group to 11.31% in the 85-year-old and above age group, reflecting the significant role of ageing as a risk factor for depression. This trend is consistent with the findings in Table 3, which shows a higher incidence of health issues among the older adult population. However, necessary underpinning is required to understand the causative factors attributing to this trend and the relationship dynamics between these variables.

Table 2 presents the prevalence of depression among the study population, taking into account mediating and moderating indicators. The result shows that respondents with physical limitations had a considerably greater prevalence of depression (11.20%) than respondents without impairments (5.05%). Similarly, the prevalence of depression was greater among those who reported social disengagement (10.60%) than those who reported social engagement (7.05%). When compared to those who said they did not feel lonely, the respondents who said they felt lonely had a higher prevalence of depression (13.73%). Furthermore, while considering the living arrangements, it was found that individuals living alone exhibited a higher prevalence of depression (9.70%) than those living with someone (7.42%).

**Table 2:** Age-sex adjusted prevalence of depression by mediating and moderating indicators in the study population in India, LASI, 20,171–8.

Indicators	Depression	95% CI	P > chi2
Functional health			0.00
No	5.05	[4.39–5.72]	
Yes	11.20	[9.84–12.6]	
Social disengagement			0.00
No	7.05	[6.22–7.87]	
Yes	10.60	[9.23–11.9]	
Feeling loneliness			0.00
No	7.21	[6.36–8.07]	
Yes	13.73	[11.8–15.6]	
Living arrangement			0.00
Living alone	9.70	[7.47–11.9]	
Living with someone	7.17	[6.27–8.07]	

*Source* computed using LASI (2017–2018) data file.

### Empirical result

Table 3 presents the odds ratios (OR) of depression based on key explanatory and socio-economic characteristics of the study population in India. Several factors showed significant associations with depression. Individuals with functional health had 1.85 times greater odds (OR: 1.85, p-value 0.001) of experiencing depression compared to those without such impairment, demonstrating that higher impairment increased the likelihood of experiencing depression. When compared to their counterparts, people who did not participate in social activities had a higher likelihood (OR 1.35, p-value 0.001) of feeling depression. The odds ratio for people who said they felt lonely was 2.51 (OR 2.51, p-value 0.001), significantly higher than those who did not report feeling lonely. When examining living arrangements, the odds of reporting lower depression was 0.91 (OR 0.91, p-value 0.001) among the respondents living with someone than those living alone. Further, considering the demographic and socio-economic factors of the respondents, higher odds of reporting depression were observed among females, currently married, educated, Muslim, SC category, richest, currently working group, and rural people.

**Table 3:** Odd ratio of depression by key explanatory and socio-economic characteristics of study population in India, LASI, 20,171–8.

Factors	Odds ratio	P > z	95% CI	
Functional health				
No				
Yes	1.85	0.00	1.58	2.17
Social disengagement				
No				
Yes	1.35	0.00	1.42	1.74
Feeling loneliness				
No				
Yes	2.51	0.00	2.14	2.65
Living arrangement				
Living alone				
Living with someone	0.91	0.00	0.84	1.10
Sex				
Female				
Male	0.55	0.00	0.89	1.11
Marital status				
Currently married				
Widowed	0.64	0.00	1.11	1.38
Others	0.75	0.09	0.69	1.29
Education				
No education				
Less than 5 years	1.11	0.18	0.92	1.24
5–9 years completed	1.37	0.00	0.88	1.15
10 years or more	1.21	0.04	0.68	0.97
Religion				
Hindu				
Muslim	1.33	0.00	0.96	1.28
Christian	0.53	0.00	0.50	0.80
Others	0.94	0.60	0.81	1.24
Caste				
Scheduled caste (SC)				
Scheduled tribes (ST)	0.39	0.00	0.36	0.53
Other backward class (OBC)	0.83	0.01	0.83	1.08
Others	0.72	0.00	0.78	1.04
MPCE quintile				
Poorest				
Poorer	0.94	0.39	0.80	1.07
Middle	0.80	0.00	0.72	0.98
Richer	1.00	0.96	0.92	1.23
Richest	1.23	0.01	1.05	1.42
Currently working				
No				
Yes	1.84	0.00	0.79	1.00
Place of residence				
Rural				
Urban	0.73	0.00	0.69	0.86
Constant	0.02	0.00	0.06	0.09
Logistic regression				
Number of observations				30,751
LR chi2(24)				678.70
Prob > chi2				0.00
Log-likelihood				− 7131.48
Pseudo R2				0.05

*Source* computed using LASI (2017–18) data file.

*Note* Chronic disease adjusted result.

**Figure 2:**
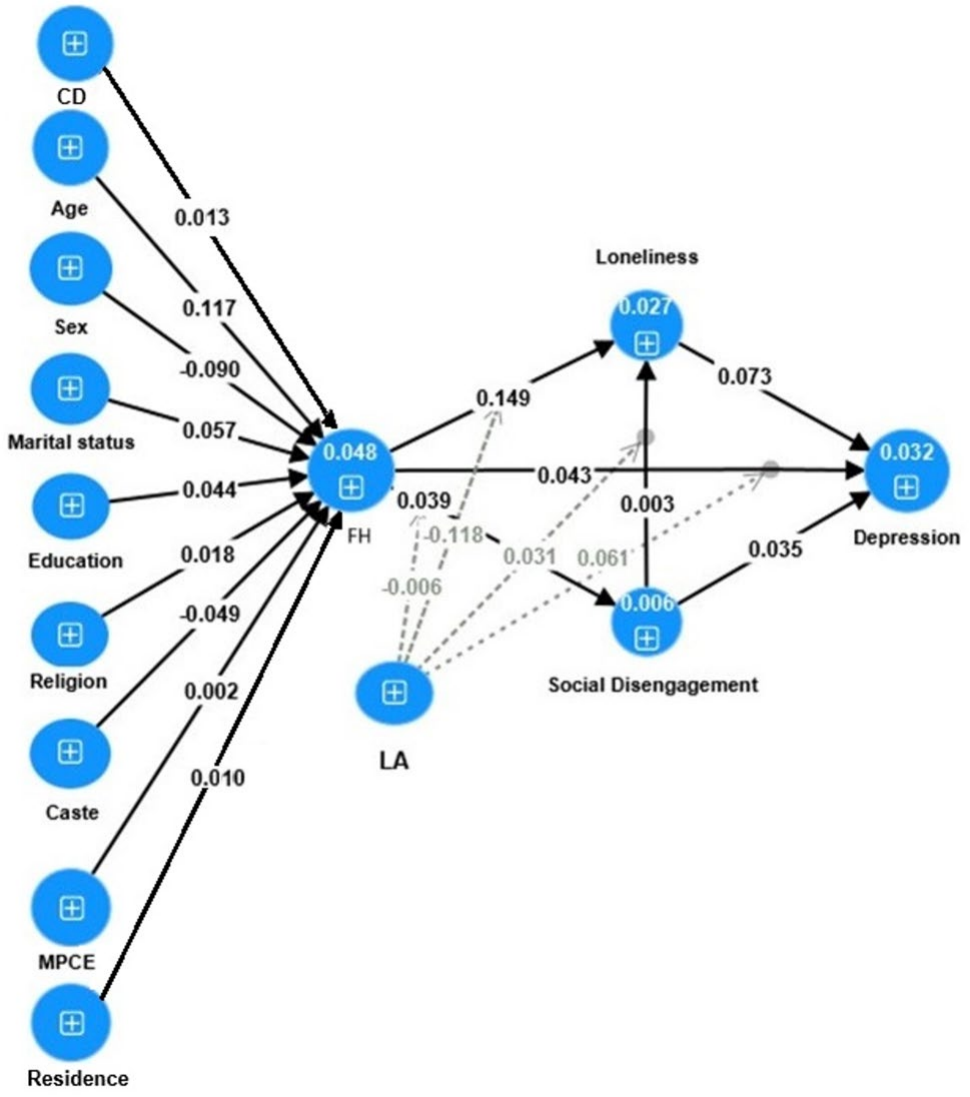
Effect of functional health and mediation effects of loneliness and social disengagement on depression and the moderation effect of living arrangement in India, LASI, 2017–18. *LA* living arrangement, *FH* functional health. The model was found to be fitted based on SRMR < 0.05 and NFI > 0.9.

In Fig. 2, the path association underlying how functional health affects depression is illustrated. The study used a PLS-structural equation model (SEM) and obtained standardised coefficients presented in Table 4. The study focused on various factors related to depression, functional health, loneliness, and personal and social life engagement and found that all these factors were significantly associated with each other. The findings revealed positive associations between depression and functional health (coefficient: 0.043), loneliness (coefficient: 0.07), and social disengagement (coefficient: 0.035). Additionally, functional health was found to be positively linked with both loneliness and social disengagement.

**Table 4:** Standardized path coefficient showing mediation effects of loneliness and social disengagement on depression and the moderation effect of living arrangement on the association between functional health and mental well-being.

Paths	Coefficients	P values		
Age → FH	0.12	0.00		
Caste → FH	− 0.05	0.00		
Education → FH	0.04	0.00		
LA → Depression	− 0.02	0.05		
LA → Loneliness	− 0.14	0.00		
LA → Social disengagement	− 0.14	0.00		
Loneliness → Depression	0.07	0.00		
Marital status → FH	0.06	0.00		
Social disengagement → Depression	0.04	0.00		
Social disengagement → Loneliness	0.00	0.46		
FH → Depression	0.04	0.00		
FH → Loneliness	0.15	0.00		
FH → Social disengagement	0.04	0.00		
Religion → FH	0.02	0.00		
Sex → FH	− 0.09	0.00		
MPCE → FH	0.00	0.33		
Residence → FH	0.01	0.04		
CD → FH	0.01	0.06		
LA × social disengagement → loneliness	0.03	0.09		
LA × FH → depression	− 0.06	0.08		
LA × FH → loneliness	− 0.12	0.00		
LA × FH → social disengagement	− 0.01	0.43		
Mediation role of social disengagement and loneliness				

	Direct effect	Indirect effect	Total effect	Indirect effect (%)
Through social disengagement	0.43	0.00	0.04	3.08
Through loneliness	0.43	0.01	0.05	22.00

*Source* computed using LASI (2017–2018) data file.

*FH* functional health, *LA* living arrangement, *CD* chronic disease.

To delve deeper into the relationships between functional health, social disengagement, and loneliness with depression, we investigated the moderating effect of living arrangements. The results showed that living arrangements had a significant moderating role, mainly when individuals were living with someone. The moderation effect of living arrangements on the relationship between functional health and depression was − 0.006. Similarly, the moderation effect of living arrangements on the relationship between functional health and loneliness was − 0.12. Furthermore, the researchers investigated the mediation effects of loneliness and social disengagement on the association between functional health and depression. The results indicated that loneliness and social disengagement partially mediated the relationship between functional health and depression, with mediation effects of 22.0% and 3.08%, respectively.

## Discussion

This study offers detailed and nuanced insights into the intricate relationship between functional health and depression among older Indian adults through the mediating role of social disengagement and loneliness and the moderating role of living arrangements using LASI, 2017–2018. Specifically, the study focuses on depression prevalence and its determinants in a diverse sample of 31,902 older adults in India. While some findings align with previous research, most results are new and original. Like previous research, our findings indicated that older individuals with functional health frequently report experiencing depression in later life^19,20,42–46^. A notable rise in the age-sex-adjusted prevalence of depression signifies growing mental health concerns among the older population in India, especially those who experience loneliness. The prevalence of depression was notably higher among the physically impaired and socially disengaged elderly population.

The study provided further support for previous research that indicates a significant correlation between physical limitations and depression. The research found that participants with functional health had almost twice the likelihood of experiencing depression compared to their counterparts. This implies that physical limitations can be a significant contributing factor to the development of depression. Physical limitation in later life hinders older people from performing their own daily living activities independently. This dependency on the other for doing these daily living activities, in turn, heightens the feeling of depression among older people^47,48^. The study’s findings highlight the importance of addressing physical limitations and their impact on mental health to help individuals better manage their overall well-being. Notably, the study clearly highlights social disengagement and loneliness as crucial factors in understanding the relationship between functional health and depression.

Results indicate that older adults who reported social disengagement and feelings of loneliness exhibited a higher prevalence of depression. Moreover, both loneliness and social disengagement partially mediated the association between functional health and depression, explaining 22.0% and 3.08% of the variance, respectively. These findings clearly indicate that social isolation and loneliness mediate the relationship between functional health and poor mental health. It highlights the importance of social disengagement and feelings of loneliness. The lack of social disengagement and feelings of loneliness limit an individual to share their feelings, including sadness or happiness. These limitations may lead to a diminished sense of purpose and belonging, critical factors for mental health. When older adults feel disconnected from others, their risk of developing depression increases, as they lack the social reinforcement that helps buffer against stress and negative emotions^49,50^. Furthermore, the poor physical limitation in later life also exacerbated social disengagement and feelings of loneliness among older people, reflecting the importance of social disengagement and feelings of loneliness in the relationship between physical limitation and depression. One possible reason may be that people with poor physical limitations often find it challenging to maintain a social network, which in turn increases social isolation, leading to higher depression^51,52^. More specifically, when physical limitation declines, the ability to maintain these relationships diminishes, leading to increased loneliness and social isolation. This lack of social interaction and support can significantly impact mental health, leading to higher prevalence rates of depression. It also somewhat confirms “the socio-emotional selectivity theory,” which talks about the physical declination with age and reduction in the social network and emotional health^53^.

It is also important to mention that the significance of living arrangements in the relationship between functional health and depression became evident from the study results. Living with someone known was associated with lower odds of depression compared to living alone among older adults. The finding was in line with previous studies, which indicated that individuals living alone were more likely to experience depressive symptoms^2,38–41^. Importantly, this study extended the existing knowledge by revealing the moderating effect of living arrangements on the relationship between functional health and depression, emphasizing the importance of its role in mitigating the adverse impact of physical health challenges on mental well-being. Furthermore, it also significantly moderated the strong relationship between functional health, social disengagement, and loneliness. The intensity of the association between functional health and depression was further moderated by living situation, which was a major moderator. This suggests that while it may be challenging to address the issues brought about by age-related physical limitations that intensify social detachment and loneliness, co-habitation with someone known may have altered this relationship, ultimately minimising the risk of depression.

Clearly, this study has important implications for public health policy and interventions aimed at improving the mental well-being of older adults. Policymakers should recognize the mediating role of social disengagement and loneliness and develop programs to encourage social engagement and reduce loneliness among older people with physical limitations. Community-based programs, social activities, and support networks can be encouraged to mitigate the adverse effects of physical constraints on mental health. Additionally, the moderating impact of living arrangements underscores the significance of considering the social environment in which older individuals reside. For those living alone, developing social networks, strengthening social security benefits, and policy formulation around social behavioural and communication changes around mental health issues among the older may offer protective benefits against depression. To safeguard the mental health of older individuals, it may be essential to implement policies that enable multigenerational living arrangements and facilitate access to social support networks.

### Limitations

It is worth mentioning that the present study had some inherent limitations. The primary concern was that it relied on cross-sectional data, making it arduous to draw causal inferences. A longitudinal study would have yielded more dependable and statistically robust conclusions. Furthermore, using PLS-SEM to handle binary outcome variables may have been less efficient than utilising interval or ratio scale information. Nonetheless, the findings of the study offer novel insights and empirical evidence that contribute to the existing research in this area.

## Conclusion and policy recommendations

In conclusion, our work offers an important new understanding of the intricate relationship between physical decline and mental health in older people. We provide insight into the potential link behind this association by highlighting the mediating roles of social disengagement and loneliness and the moderating impact of living arrangements. These results provide a basis for developing targeted interventions to improve older persons’ overall quality of life and mental health. Resilience and mental wellness in ageing populations may be fostered by public health programs that prioritise encouraging social engagement, lowering loneliness, and facilitating multigenerational living arrangements. It is necessary to conduct more studies to examine such interventions’ long-term results and efficacy in enhancing mental health.

## Data and methodology

We utilised secondary data from the Longitudinal Ageing Study in India (LASI) 2017–2018. It is a nationally representative large-scale survey of older individuals in India, which was carried out by the International Institute for Population Sciences (IIPS) in collaboration with the Harvard School of Public Health, the University of Southern California, and the University of Maryland. It used a three-stage sampling method for rural areas and a four-stage sampling method for urban areas. In the first step, primary sampling units (PSUs) or subdistricts (Tehsils/Talukas) were chosen using explicit and implicit stratification. Explicit stratification entailed grouping subdistricts into homogeneous strata according to a number of factors, such as the size of the population, the percentage of Scheduled Caste/Scheduled Tribe members, and the employment of men in non-agricultural industries. Subdistricts within a stratum were organized according to female literacy rates through implicit stratification. Using probability proportionate to size (PPS) sampling, 456 PSUs were chosen. The second and third stages encompassed the selection of secondary sampling units (SSUs), which are villages or census enumeration blocks (CEBs). An additional stage was included for urban areas, involving the random selection of one CEB from each SSU. The third and fourth stages involved the selection of households (HHs) from villages/CEBs. It covered 31,913 individuals aged 60 and above. For more information, please refer to the report^6^.

### Measurement

#### Outcome variable

##### Depression

The Composite International Diagnostic Interview (CIDI) is a widely recognised instrument utilised by clinicians and researchers to evaluate the presence of major depression in both clinical and epidemiological settings^54,55^. It determines a probable psychiatric diagnosis of major depression and has been validated in field settings and population-based health surveys. LASI provided information regarding the Short Form Composite International Diagnostic Interview (CIDI-SF) to assess the prevalence of major depression based on diagnostic symptoms. The three-step screening approach used by LASI included questions about dysphoria experienced for at least two weeks during the previous 12 months, using the phrases “most of the day” and “almost daily.” In addition, seven questions with good reliability (Cronbach alpha = 0.72) evaluated signs of depression, such as weariness and loss of interest. A depression severity scale (0–10) was created using the CIDI–SF version, with a score of three or higher denoting significant depression and a score of three or lower denoting non-existence.

#### Key independent variable

##### Functional health

Functional health (FH) was measured using the Activities of Daily Living (ADL) and Instrumental Activities of Daily Living (IADL) scales, which have been widely utilised for measuring functional health among older people^56–58^. In LASI, respondents were asked about the level of difficulty they experienced in performing daily activities over the past three months. Under the ADL and IADL, it asked about whether feeling difficulty in activities such as *“dressing; walking across a room; bathing; eating; getting in or out of bed; using the toilet, including getting up and down; preparing hot meals (cooking and serving); grocery shopping; making telephone calls; taking medications; doing household or gardening tasks; and managing finances, including paying bills and tracking expenses*”. To indicate whether respondents had trouble with each activity, participants were given yes/no binary answers. Cronbach’s alpha was used to assess the ADL and IADL scales’ reliability, and the resultant value of 0.88 indicated good scale reliability. Furthermore, a score of 1 denoted functional health if any ADL or IADL question received a “yes”; a score of 0 indicated no functional health for all “no” responses.

#### Feeling of loneliness

We measured the feeling of loneliness using a single-item scale. Many previous research has also employed the single-item scale, as evidenced in studies by Zong et al.^59^, Tan et al.^60^, and Bai et al.^61^. In LASI, respondents were required to rate how often they felt alone in the past week, with responses ranging from 1 (meaning rarely or never, i.e., less than one day) to 4 (meaning most or all of the time, i.e., 5–7 days). The single-item question is from the CES-D-10, a 10-item depression scale developed by the Centre for Epidemiological Studies. Using the information, we further established a binary variable where a score of 1 was assigned to individuals categorised as “lonely” (comprising individuals who reported feeling lonely often, most or all the time, or sometimes), and a score of 0 was assigned to individual categorised as “not lonely” (comprising individuals reported feeling lonely rarely or never), as defined in previous studies by Holmén and Furukawa^62^, Tiikkainen and Heikkinen^63^, and Nicolaisen and Thorsen^64^.

#### Social disengagement

*S*ocial disengagement (SD) was operationally defined as disengagement from participation in different social activities, including any event and ceremony, club or organisation, friend, and leisure activity. LASI collected information on cultural involvement, dining out, participation in indoor and outdoor games, attending religious functions and political meetings, reading news, books, papers, and magazines, watching TV, listening to the radio, using a computer for email and net surfing, and membership in social organisations (daily to never). Good reliability was shown by all of the questions (Cronbach’s alpha = 0.67). Using these indicators we measured one’s social disengagement (Ajaero et al., 2016; Somefun et al., 2019; Dhawan et al., 2023; LASI, 2020 report). Responses were converted into binary data, with activities being classified as “yes” if they participated once a month to never or not relevant, and “no” if they participated daily to several times a month. A value of 1 was given to respondents who answered “no” to every question as socially disengaged; otherwise, it was 0.

#### Living arrangements

Living arrangements refer to the household’s composition and structure, comprising the number of household members and their relationships with each other^65^. It may include living alone, with a spouse, with a spouse and children^6^. Respondents to the LASI survey were asked about the total number of people living in their home, including family members, domestic helpers, children, newborns, and anyone temporarily away from it, such as those seeking medical attention or visiting family, as well as their relationships. We operationally established a variable for living arrangements using this information, which was split into “living alone” (1) and “living with someone” (0). The latter group included living together with a spouse, adult child, parent, or both, or at least with any member.

#### Demographic, social, and economic characteristics

To ensure a comprehensive analysis, we considered various demographic, social, and economic factors that could impact the outcome variables. These factors included age (categorised as 60–64, 65–69, 70–75, and 75 + with all respondents aged 75–116 included for robust analysis as depression prevalence showed less variation after age 75, for further details, see Appendix 1), gender (male/female), education level (no schooling, 1–5 years completed, 5–9 years completed, and 10 + years completed), religious affiliation (Hindu/Muslim/Christian and other), social category (scheduled caste/scheduled tribe/other backward class/other), monthly per capita expenditure (MPCE) quintile (poor to rich), current employment status (yes/no), and place of residence (rural/urban). This allowed us to obtain an accurate understanding of the adjusted effect of the critical indicators we selected. Since chronic health conditions such as coronary heart disease (heart attack or Myocardial Infarction), congestive heart failure, or other chronic heart problems, Stroke, Arthritis or rheumatism, Osteoporosis or other bone/joint diseases, Any neurological, or psychiatric problems such as depression, Alzheimer’s/Dementia, unipolar/bipolar disorders, convulsions, Parkinson’s, etc., and High cholesterol have their own effect on the functional and mental health, we considered these chronic diseases (diagnosed in at least one/ none of these conditions) to obtain the adjusted effect.

### Statistical approach

In this paper, we employed bivariate analysis and logistic regression. Given the dichotomous nature of the response variable, logistic regression was found to be the most appropriate method. Further, age offset was incorporated to account for the observed relationship between age and depression to uphold fairness and comparability across factors. This involved controlling for the effect of age on the relevant variables and exposing each participant to the same amount of information. Further, we employed a robust regression approach to deal with outliers in the age data. Using the national age-sex composition, we adjusted the prevalence of depression according to the respondents’ age and sex using national age-sex composition to further improve our study.

Additionally, we examined moderation and mediation effects using partial least squares structural equation modelling (Pls-SEM), commonly used in social sciences, psychology, and public health. We employed the PLS algorithm to investigate the mediating effects of loneliness and social disengagement between functional health and depression. It also allows us to handle non-linear data characteristics of binary variables. We employed PLS-logit, as recommended by previous studies, to account for the binary nature of the outcome variable^66,67^. Given the condition of the study’s variables, which lacked a distinct distributional structure, we further employed the bootstrap estimation approach using 5000 samples to draw reliable inferences. With the help of this resampling technique, we generated a distribution of indirect effect estimates and precisely calculated their significant level. As a result of the product of direct effects, indirect impact estimates frequently exhibit non-normal distributions, according to Zhang et al.^68^. Researchers have recommended the bootstrap strategy for obtaining the most accurate indirect effects when there is a non-normal distribution^34,68^. All the analyses were adjusted by the chronic health conditions. In the current study, we used Stata version 17 and SmartPLS version 4, two widely used research software programs, to analyse the complex nexus between variables.

### Supplementary Information

Supplementary Information.

## Data Availability

Authors do not have the right to share the data. However, the data can be obtained at a reasonable request from the International Institute for Population Sciences, India. Interested parties may obtain the data by submitting a formal application form to the institute. Access to the data is facilitated through a straightforward application process available via the institute’s website at https://www.iipsindia.ac.in/content/lasi-wave-i.

## Abbreviations

FH: Functional health
ADL: Activities of daily living
IADL: Instrumental activities of daily living
LA: Living arrangement
LASI: Longitudinal ageing survey of India
OBC: Other backward class
OR: Odds Ratios
PLS-SEM: Partial least square structural equation model
SC: Scheduled caste
SD: *S*Ocial disengagement
ST: Scheduled tribe
CD: Chronic diseases
