# Supplementary material for: Mediating role of social disengagement and loneliness in the nexus between functional health and mental well-being in older individuals
Source: Sci Rep. 2024 Jul 14;14:16232. doi: 10.1038/s41598-024-66919-9 (PMC11247097; doi:10.1038/s41598-024-66919-9)
Supplement: Supplementary file 1 — Supplementary Information. [file 41598_2024_66919_MOESM1_ESM.docx]

## **APPENDIX**

Appendix 1 Sex-adjusted depression distribution by age of respondent

**Appendix 2. Age-sex adjustment**

We used both age and sex to examine the prevalence at the national level. Basically, national age-sex composition helps to adjust the potential difference and exposure of individual from different age and sex. Since, prevalence and exposure of individual from different age to being depressed are different and it is also varied by male and female, it is always necessary to examine prevalence after adjusting age and sex. We estimated logistic regression to get the age-sex adjusted prevalence of depression at the national level. We grouped each individual into different age groups and utilised sample weight. Then, average predicted probability was generated at the national and different category level to obtain national level prevalence of depression.
